# Causal relationship between metabolites, cerebral volume, and amyotrophic lateral sclerosis: A 2-step Mendelian randomization analysis

**DOI:** 10.1097/MD.0000000000050769

**Published:** 2026-09-18

**Authors:** Youjia Liu, Sihui Chen, Yanyun Wu, Wenting Zhou, Huifang Shang, Ruwei Ou

**Affiliations:** a Department of Neurology, West China Hospital, Sichuan University, Chengdu, Sichuan, China.

**Keywords:** amyotrophic lateral sclerosis, causal inference, cerebral volume, genome-wide association study, Mendelian randomization, metabolomics, neurodegeneration, plasma metabolites

## Abstract

Amyotrophic lateral sclerosis (ALS) is a progressive neurodegenerative disorder characterized by motor neuron degeneration. Although metabolic abnormalities have been implicated in ALS, the causal relationships between circulating metabolites and ALS remain unclear. This study aimed to investigate the potential causal associations between plasma metabolites and ALS risk and to explore the mediating role of cerebral volume. A 2-sample Mendelian randomization (MR) study was conducted using publicly available genome-wide association study summary statistics. Genetic instruments for plasma metabolites were obtained from metabolomic GWAS datasets, and ALS-associated genetic data were used as the outcome. The inverse-variance weighted method was used as the primary analysis, with sensitivity analyses performed using MR-Egger regression, weighted median analysis, MR-PRESSO, heterogeneity testing, and leave-one-out analysis. A 2-step MR analysis was further conducted to evaluate whether cerebral volume mediated the associations between metabolites and ALS. Six metabolites showed significant associations with ALS risk. Genetically predicted higher levels of *N*,*N*-dimethylalanine, Behenylcarnitine (C22), and deoxycarnitine were associated with increased ALS risk, whereas higher levels of (N(1) + N(8))-acetylspermidine, Betaine, and tetradecadienedioate (C14:2-DC) were associated with decreased ALS risk. Sensitivity analyses supported the robustness of the findings. Two-step MR analysis suggested that cerebral volume did not significantly mediate the associations between these metabolites and ALS risk. This study identified 6 circulating metabolites genetically associated with ALS risk. These findings provide insights into the potential metabolic pathways involved in ALS and suggest that the observed associations are unlikely to be mediated through cerebral volume changes.

## 1. Introduction

Amyotrophic lateral sclerosis (ALS) is a severe neurodegenerative disorder that results in the gradual deterioration and loss of motor neurons. This progression causes symptoms such as muscle weakness, atrophy, and stiffness. Ultimately, the disease impairs respiratory and swallowing functions, significantly affecting patient survival and quality of life.^[1]^ The pathogenesis of ALS remains incompletely understood. However, research suggests that genetic factors, oxidative stress, excitotoxicity, neuroinflammation, and mitochondrial dysfunction may play an important role in the development of the disease.^[2]^ The annual incidence of ALS is about 1 to 2 cases per 1,00,000 individuals, with a higher prevalence in males than in females. Although no cure exists, available medications and supportive therapies can help alleviate symptoms and improve patients’ quality of life. Recent advances in understanding ALS pathophysiology have led to novel therapeutic strategies and experimental drugs, offering new hope for patients.

Metabolites are intermediates or end products of metabolic reactions in organisms, and their levels are influenced by multiple factors, including genes, diet, lifestyle, intestinal microbiota, and disease.^[3-5]^ Based on their chemical properties and biological functions, metabolites can be systematically classified into several categories: lipids, amino acids, carbohydrates, nucleotides, cofactors, and vitamins. These small molecules play an important role in biological processes such as energy metabolism, cellular signaling, oxidative defense mechanisms, and programmed cell death. Through metabolomic techniques, researchers can comprehensively characterize and quantify metabolites in biological samples to identify biomarkers associated with specific diseases so they can influence disease risk and become targets for therapeutic intervention.^[4]^

Metabolites play a crucial role in both health and disease, with alterations in their concentration being closely linked to the onset and progression of neurodegenerative diseases, such as Alzheimer disease and Parkinson disease. Genome-wide association studies (GWAS) and whole-exome sequencing studies have identified numerous genetic loci and functional variants related to metabolite levels, indicating the significant contribution of genetic factors in metabolic regulation. The high heritability observed for many metabolites^[3,6]^ makes them particularly suitable for Mendelian randomization (MR) studies. While randomized controlled trials (RCTs) could theoretically establish causal relationships for metabolites, such studies face substantial challenges. RCTs are typically expensive and time-consuming to conduct, and critically, the random allocation of metabolite components is often impractical in real-world settings. MR offers a powerful alternative for causal inference by utilizing genetic variants as instrumental variables (IVs) to examine the relationship between exposure factors (metabolites) and disease outcome.^[7]^ This approach benefits from the natural randomization of alleles during conception, effectively minimizing confounding by most risk factors and reducing potential bias in outcome assessment. The availability of large-scale GWAS data for both metabolites and ALS now enables MR analyses with significantly enhanced statistical power.

In this study, we conducted a 2-sample MR analysis to explore the causal relationship between 1091 blood metabolites and 309 metabolite ratios (which cover 8 major classes of metabolites, namely lipids, amino acids, exogenous substances, nucleotides, coenzymes and vitamins, carbohydrates, peptides, and energy substances) and ALS risk. Given the close relationship between cerebral volume and ALS,^[8-10]^ we further performed a 2-step MR analysis to explore whether the effects of the identified metabolites on ALS are mediated by regulating cerebral volume.

## 2. Materials and methods

### 2.1. Research overview

**Figure 1.**
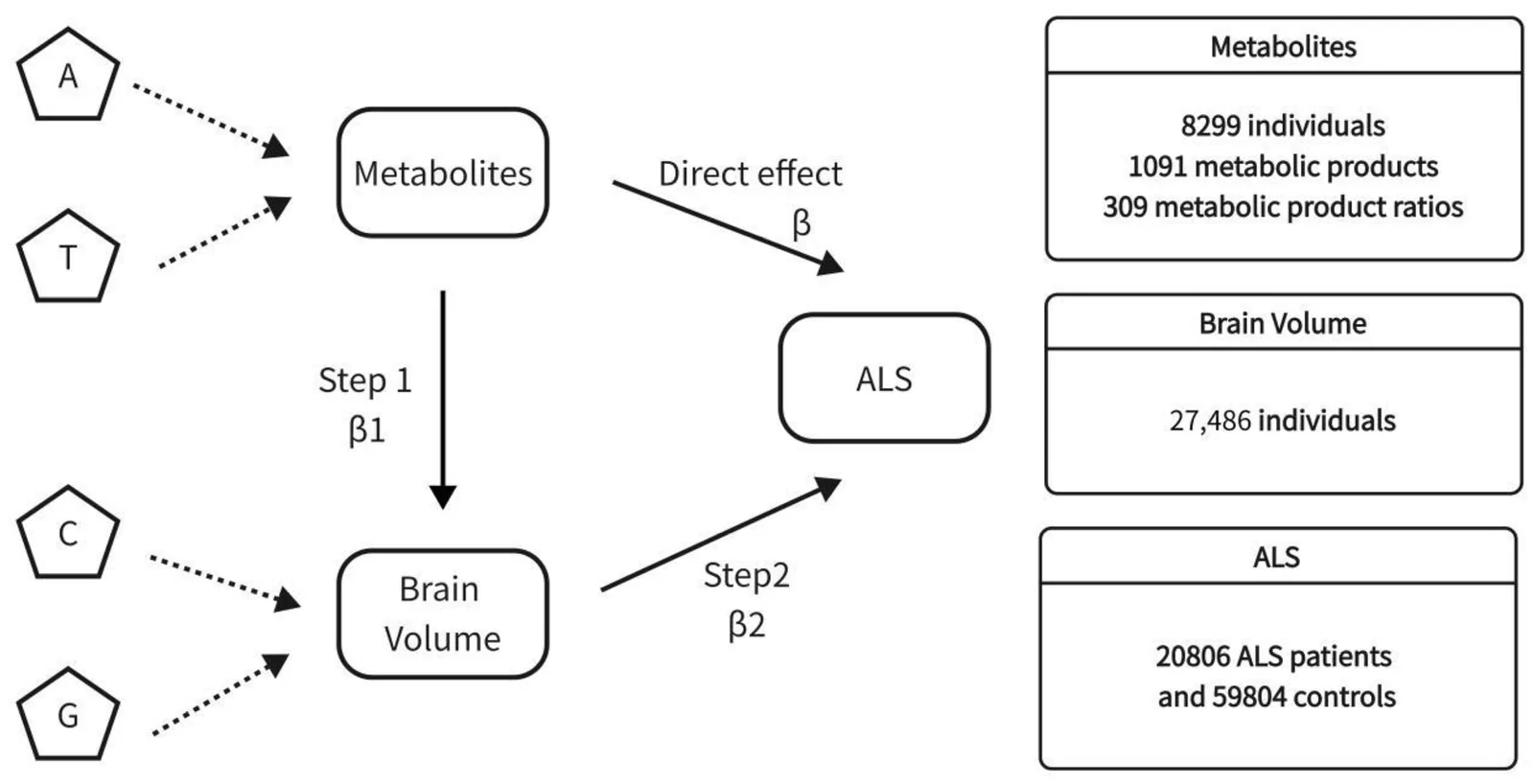
Two-step MR analysis process. ALS = amyotrophic lateral sclerosis, MR = Mendelian randomization.

A schematic summary of the research methodology is illustrated in Figure 1. The analysis began by employing single nucleotide polymorphisms (SNPs) derived from aggregated datasets as genetic markers for risk factors. We then conducted a 2-sample MR approach to evaluate potential causal relationships between metabolites and ALS. MR analysis was implemented to investigate potential mediation effects and examine whether cerebral volume is an intermediary in the metabolic pathways associated with ALS risk. All data were sourced from publicly available repositories, with proper ethical clearances secured for the original studies contributing to these pooled datasets.

### 2.2. Data sources

#### 2.2.1. Metabolite

The findings originate from an extensive GWAS conducted within the Canadian Longitudinal Study on Aging (CLSA). Researchers systematically analyzed genetic data and plasma metabolite profiles from 8299 European-ancestry participants, examining 1091 distinct blood metabolites and 309 metabolite ratios in total.^[11]^ This represents one of the most extensive metabolomic investigations to date. Using ultra-high-performance liquid chromatography-tandem mass spectrometry (UPLC-MS/MS), the team precisely measured metabolite concentrations and correlated these with genome-wide genotyping data. They cross-referenced these findings with genome-wide genotyping data to pinpoint genetic variations linked to metabolite levels. The investigation discovered 690 metabolite-gene connections across 248 genetic spots and 143 metabolite ratios tied to 69 genetic regions. This trove of information serves as a goldmine for delving into how metabolites influence human health and disease.

#### 2.2.2. Cerebral volume

A GWAS conducted in the UK Biobank (UKB) initiative identified genetic variants linked to cerebral volume. This large-scale analysis involved 27,486 participants and focused specifically on variations in total cerebral volume.^[12]^ The study pinpointed key variants that influence this trait by examining the genetic underpinnings of brain size. These findings serve as a valuable foundation for further research into the hereditary factors shaping brain structure.

#### 2.2.3. Amyotrophic lateral sclerosis

Park et al conducted a landmark study that systematically investigated ALS susceptibility through an integrative analysis of GWAS data and transcriptomic profiles.^[13]^ By analyzing a massive dataset of 20,806 ALS patients and 59,804 controls of European descent, coupled with transcriptomic profiling across 19 neural and hematological tissue types, the team pinpointed 27 genes with significant ALS associations, including 7 novel genetic risk loci that had not been previously reported in ALS pathogenesis. This comprehensive approach, merging large-scale GWAS meta-analysis with multi-tissue expression data, yielded novel insights into ALS pathogenesis. The discovery of these genes shed fresh light on the genetic underpinnings of ALS, while conditional and functional enrichment analyses reinforced their autonomy and biological relevance. Leveraging these robust GWAS findings, the study establishes a strong genetic foundation for investigating potential causal links between ALS and other factors.

### 2.3. Genetic instruments selection

#### 2.3.1. Selecting genetic instruments

The genetic instrumental variables must adhere to 3 key assumptions^[14]^: Genetic variants must exhibit a robust association with exposure determinants; genetic variants must remain independent of possible confounding factors; and genetic variations must not independently impact outcomes outside the context of exposure variables. We eliminated variants in the GWAS dataset with minor allele frequencies below 0.01. We applied the clumping feature in PLINK software to pinpoint individual SNPs for each exposure factor, relying on European data from the 1000 Genomes Project as our benchmark.^[15]^ We applied strict clumping criteria to identify significant genetic variants, including an *r*^2^ threshold of <0.001, a 10,000 kb window, and a *P*-value cutoff of 5 × 10^−8^. However, for metabolite-related SNPs, we adopted a more lenient *P*-value threshold of 1 × 10^−5^, consistent with prior Mendelian randomization research.^[16-18]^ This adjustment was justified because SNPs below this threshold accounted for the highest variance in microbial traits,^[19]^ suggesting stronger biological relevance due to their robust association with the target phenotype. The finalized exposure factor SNPs, post-clumping, are detailed in Table S1, Supplemental Digital Content 1.

#### 2.3.2. Removing confounders

To sidestep any possible confounding variables, we excluded SNPs strongly linked to these factors among European subjects in the PhenoScanner database.^[20]^ We considered a range of potential confounders, such as dietary habits, diabetes, drinking patterns, and smoking habits. These traits impact metabolite profiles^[21,22]^ and the likelihood of developing ALS.^[23-26]^ After removing these confounders, the remaining exposure factor SNPs are detailed in Table S2, Supplemental Digital Content 2.

#### 2.3.3. Quality control of genetic instruments

We filtered out palindromic SNPs exhibiting allele frequencies exceeding 0.42 to minimize potential strand-flipping issues. Furthermore, to enhance the reliability of our genetic instrumental variables, we eliminated polymorphic anomalous SNPs identified through RadialMR,^[27]^ thereby strengthening the overall validity of our analysis. RadialMR detects atypical SNPs exhibiting polytropic patterns by employing a dissimilarity test, specifically a modified *Q* statistic, with a significance threshold of 0.05. To evaluate the robustness of our genetic instrumental variables, we determined their *F* statistics, including only those with an *F* value exceeding 10 in the MR analysis. The remaining genetic instruments’ statistical power was then estimated following Burgess’s method, which indicated a power of 1.^[28]^ Post-exclusion of confounders, a minimum of 3 SNPs were preserved within each category upon finalizing quality checks, facilitating MR studies.

## 3. Statistical analyses

### 3.1. Two-sample MR analysis

Investigations were carried out to determine if there is a causal relationship between metabolites and ALS and if ALS has a causal impact on the production of metabolites. We relied on the inverse variance weighting technique for the primary causal analysis, which is grounded in a multiplicative random effects model.^[29]^ Given the potential for IVW estimates to be skewed when employing multinomial instrumental variables,^[30]^ we opted to employ 3 supplementary approaches concurrently to ascertain causality, thereby bolstering the dependability of our findings (Table S3, Supplemental Digital Content 3). These additional methods encompass the weighted median,^[31]^ the weighted plurality,^[32]^ and the MR-Egger^[33]^ techniques. The study presents the conclusive findings from the inverse variance-weighted (IVW) analysis. We adjusted the *P*-values obtained from the MR using the Benjamini-Hochberg false discovery rate procedure to account for multiple comparisons across different strata. An adjusted *P*-value threshold of .05 was established to determine statistical significance (Table S5, Supplemental Digital Content 4). However, the multiple testing correction may have been overly stringent given the extensive number of categorical variables and their nested relationships in this investigation.

### 3.2. Multiple validity testing

This study employed a methodical strategy to evaluate pleiotropy, strengthening the reliability of our causal conclusions. We conducted a pleiotropy assessment for every metabolite involved in the study, leveraging genetic variant associations across various traits and conditions. The goal was to pinpoint genetic variants influencing multiple outcomes, uncovering any underlying pleiotropic effects that could skew interpretations.

### 3.3. Leave-one-out

Our study employed a leave-one-out approach to evaluate each SNP’s influence on the findings. The data revealed that causal effect estimates maintained statistical significance across all iterations, demonstrating that no individual SNP disproportionately impacted the results. These robust findings indicate that our conclusions are not driven by any specific genetic marker, reinforcing the validity of our causal inference. The consistency of these outcomes across multiple tests underscores our analysis’s methodological strength and reliability.

### 3.4. Mediation analysis

To investigate whether the influence of specific metabolites on ALS operates via changes in cerebral volume, we conducted a 2-stage MR analysis. The initial phase involved determining the causal impact of these metabolites on cerebral volume measurements. Subsequently, we examined whether variations in cerebral volume had a causative effect on ALS risk. This approach allowed us to evaluate the potential mediating role of brain structure in the metabolite-ALS relationship. In studying the subtle influence of metabolites on ALS, we delved into the relationship with cerebral volume through a method known as the coefficient product technique.^[34]^ This essentially multiplies the impact of each step in the analysis, yielding a figure for the indirect effect, or what we call β, which is the product of β1 and β2. To pinpoint how much the specific metabolites contribute to the overall effects of ALS on cerebral volume, we divided the indirect effect by the total impact. We also utilized the Delta method^[35]^ to calculate the standard errors of the indirect effects.

### 3.5. Analytical tools

Data analysis was conducted using the R programming language and the software packages Two-Sample MR (0.6.8), MR-PRESSO (1.0), and RadialMR (1.1).

### 3.6. Ethics approval and consent to participate

This study used publicly available GWAS summary statistics. Since no individual-level participant data were accessed or collected, additional ethical approval and informed consent were not required. The original GWAS studies obtained ethical approval from their respective institutional review boards.

## 4. Results

### 4.1. Causal effects of metabolites on ALS

Our findings demonstrated compelling causal links between multiple metabolites and ALS development (Table S4, Supplemental Digital Content 5). Using IVW analysis, we identified 6 metabolites showing statistically significant associations with ALS (*P* < .05), comprising an equal distribution of 3 amino acids and 3 lipid compounds. Crucially, despite adjustment for false discovery rate, 6 metabolite genetic predispositions remained statistically significant (Table S5, Supplemental Digital Content 4).

Our systematic evaluation of metabolic profiles revealed multiple metabolites demonstrating statistically significant associations with ALS risk. We disclosed considerable correlations between these metabolites and ALS. Levels of *N*,*N*-dimethylalanine (OR = 1.146, 95% CI: 1.001–1.313, *P* = .048), Behenylcarnitine (C22) (OR = 1.128, 95% CI: 1.004–1.267, *P* = .043) and deoxycarnitine (OR = 1.186, 95% CI: 1.005–1.400, *P* = .043) had a risk effect for ALS, whereas (N(1) + N(8))-acetylspermidine (OR = 0.876, 95% CI: 0.789–0.972, *P* = .021), Betaine (OR = 0.807, 95% CI: 0.679–0.958, *P* = .021) and Tetradecadienedioate (C14:2-DC; OR = 0.794, 95%CI: 0.679–0.928, *P* = .011) were associated with a decreased risk of ALS (Table 1). Figure 1 displays a forest plot summarizing the estimated effects of SNPs on the specified metabolites, along with their corresponding 95%CI. Additionally, the figure includes a scatterplot comparing SNP effects for the 2 metabolites against ALS risk, with colored trend lines representing various MR analyses. The visualization also presents forest plots showing individual and aggregated SNP effect sizes derived from MR estimates.

**Table 1:** Statistically significant metabolites of this study.

Exposure	Number of SNPs	*P* value	OR (95% CI)
Amino acid			
(N(1) + N(8))-acetylspermidine	3	.000	0.876 (0.789–0.972)
Betaine levels	3	.000	0.807 (0.679–0.958)
*N,N*-dimethylalanine	3	.048	1.146 (1.001–1.313)
Lipid			
Tetradecadienedioate (C14:2-DC)	3	.000	0.794 (0.679–0.928)
Behenylcarnitine (C22)	3	.043	1.128 (1.004–1.267)
Deoxycarnitine	3	.043	1.186 (1.005–1.400)

CI = confidence interval, OR = odds ratio, SNP = single nucleotide polymorphism.

**Figure 2.**
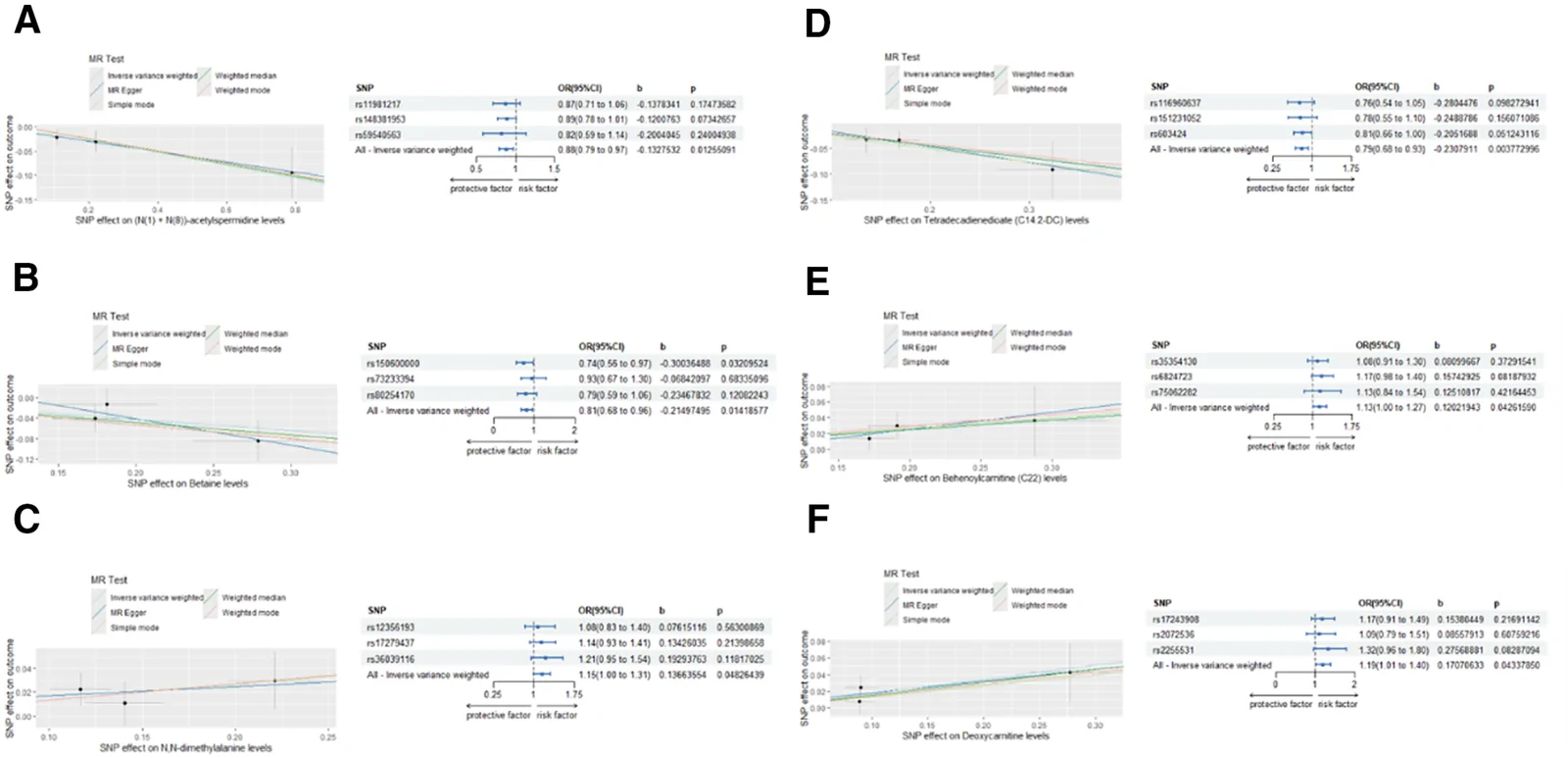
MR plot of metabolite and ALS relationship. (A) (N(1) + N(8))-Scatterplot of SNP effects of acetylspermidine levels versus ALS, with the slope of each line corresponding to the estimated MR effect of each method; (N(1) + N(8))-Forest plots of MR-estimated effect sizes of individual and combinations of SNPs for the effect of acetylspermidine levels on ALS. (B) Scatter plots of SNP effects of Betaine levels versus ALS, and forest plots of MR-estimated effect sizes of individual and combined SNPs for the effect of Betaine levels on ALS. (C) Scatterplot of SNP effects of *N,N*-dimethylalanine levels versus ALS; and forest plot of MR-estimated effect sizes of individual and combined SNPs for the effect of *N,N*-dimethylalanine levels on ALS. (D) Scatterplot of SNP effects of Tetradecadienedioate (C14:2-DC) levels versus ALS; and forest plot of MR-estimated effect sizes of individual and combined SNPs for the effect of Tetradecadienedioate (C14:2-DC) levels on ALS. (E) Scatterplot of SNP effects of Behenylcarnitine (C22) levels versus ALS; and forest plot of MR-estimated effect sizes of individual and combined SNPs for the effect of Behenylcarnitine (C22) levels on ALS. (F) Scatterplot of SNP effects of deoxycarnitine levels versus ALS; and forest plot of MR-estimated effect sizes of individual and combined SNPs for the effect of deoxycarnitine levels on ALS. ALS = amyotrophic lateral sclerosis, CI = confidence interval, MR = Mendelian randomization, OR = odds ratio, SNP = single nucleotide polymorphism.

Following rigorous screening procedures, we identified 3 SNPs significantly associated with each of the 6 metabolite classes (Fig. 2). These selected SNPs were subsequently employed in our MR analysis. Sensitivity analyses demonstrated the absence of horizontal pleiotropy through pleiotropy tests (Table S6, Supplemental Digital Content 6), the robustness of MR results as confirmed by leave-one-out analysis (Table S7, Supplemental Digital Content 7), and no significant heterogeneity among the selected SNPs for all metabolites (Table S8, Supplemental Digital Content 8).

### 4.2. Mediation analyses

**Figure 3.**
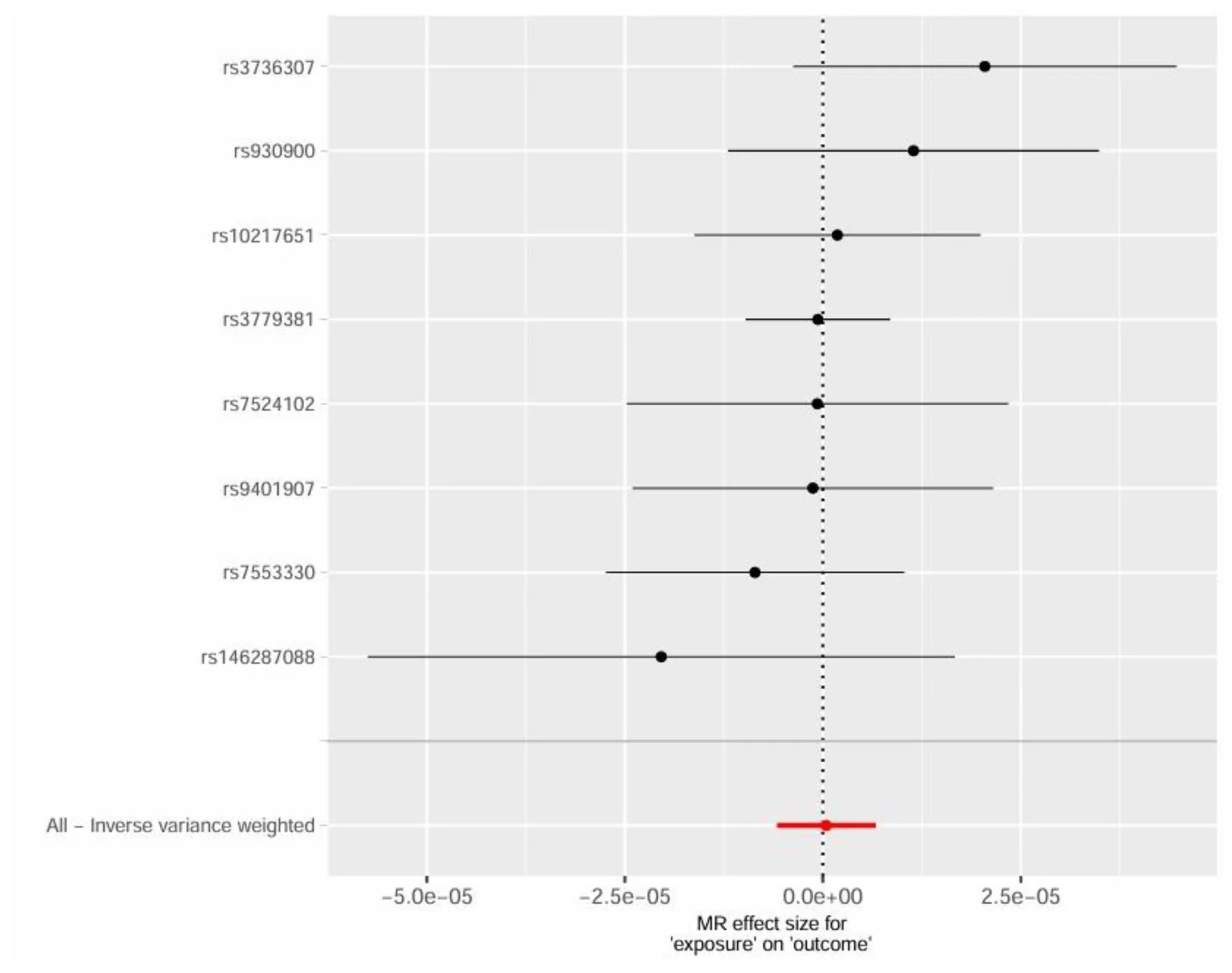
MR analysis results of cerebral volume and ALS. ALS = amyotrophic lateral sclerosis, MR = Mendelian randomization.

We used SNPs for these 6 metabolites to estimate the causal effects of these metabolites on cerebral volume. We found that none of these 6 metabolites was causally associated with increased cerebral volume, and the *P*-values for all metabolites were >.05 (Table 2). Subsequently, we used SNPs associated with cerebral volume to assess the causal effect of cerebral volume on ALS. We found that metabolites with significant effects on ALS were not mediated through cerebral volume (β = 4.415 × 10^−7^; *P* = .889; Fig. 3). These findings suggest that the associations between these 6 metabolites and ALS are unlikely to be mediated through cerebral volume. We conducted a 2-way analysis to further explore the relationship between cerebral volume and ALS. ALS has a strong causal effect on cerebral volume (β = −1158.01, 95% CI: −1454.46 to −861.56, *P* = 1.92 × 10^−14^).

**Table 2:** MR analysis results of metabolites and cerebral volume.

Metabolites	Number of SNPs	*P*
(N(1) + N(8))-acetylspermidine	3	.663
Tetradecadienedioate (C14:2-DC)	2	.140
Betaine	3	.988
*N,N*-dimethylalanine	3	.440
Behenylcarnitine (C22)	3	.816
Deoxycarnitine	3	.428

MR = Mendelian randomization, SNP = single nucleotide polymorphism.

## 5. Discussion

This study employed 2-sample MR analysis using pooled data from the largest metabolite and ALS GWAS to investigate causal relationships between metabolites and ALS risk, revealing that elevated levels of *N*,*N*-dimethylalanine, Behenylcarnitine (C22), and deoxycarnitine increased the risk of ALS. At the same time (N(1) + N(8))-acetylspermidine, Betaine, and Tetradecadienedioate (C14:2-DC) exert protective effects, with mediation analysis demonstrating that these associations operate independently of cerebral volume changes despite the observed significant reduction in cerebral volume among ALS patients, thereby providing potential intervention targets for ALS risk reduction and disease management.

In the current study, 3 metabolites were observed to have protective effects on the risk of ALS: (N(1) + N(8))-acetylspermidine, Betaine, and C14:2-DC. (N(1) + N(8))-acetylspermidine is a polyamine metabolite formed by the acetylation of spermidine. Polyamines are important in cell growth, proliferation, differentiation, and stress response.^[36]^ Its potential protective effect on ALS pathogenesis may be through mechanisms involving antioxidant and anti-apoptotic pathways.^[37-39]^ Betaine is an important nutrient with multiple biological activities, such as antioxidant, anti-inflammatory, and methylation regulation. It may have a potential protective effect on the pathogenesis of ALS, which may be related to the regulation of oxidative stress, inflammatory reactions, and apoptosis.^[40,41]^ Betaine also plays an important role in regulating the interaction between the intestinal microbiota and host health. This regulation may affect the development of neurodegenerative diseases.^[42,43]^ C14:2-DC is a long-chain dicarboxylic acid, a fatty acid metabolite involved in cell membrane phospholipid synthesis, energy production, and cell signaling.^[44,45]^ Elevated levels are associated with a reduced risk of ALS. They may play a protective role in the pathology of ALS through mechanisms related to the regulation of oxidative stress or participation in neuroinflammatory processes.^[43,46,47]^ These results provide a theoretical basis for the protective effects of (N(1) + N(8))-acetylspermidine, Betaine, and C14:2-DC in ALS. Although there is currently limited evidence directly linking these metabolites to the protective effects of ALS, their potential neuroprotective mechanisms warrant further study.

On the contrary, 3 metabolites, including *N*,*N*-dimethylalanine, Behenylcarnitine (C22), and deoxycarnitine were observed to be significantly associated with an increased risk of ALS. *N*,*N*-dimethylalanine is a dimethylated amino acid derived from alanine that may affect neurological health by participating in cell signaling, stress responses, and energy metabolism.^[43,48]^ Its levels are positively correlated with the risk of ALS, suggesting that it may be involved in the pathogenesis of ALS.^[49-51]^ Behenylcarnitine (C22) is an important lipid carrier in fatty acid metabolism and is closely related to energy metabolism.^[52,53]^ Elevated levels are associated with an increased risk of ALS, which may affect the pathogenesis of ALS by affecting energy metabolism and oxidative stress responses.^[54]^ The carnitine derivative Deoxycarnitine is involved in energy metabolism and fatty acid oxidation,^[55]^ and its role in ALS has been poorly studied. However, serum Deoxycarnitine levels in patients with ALS may be related to disease progression and severity,^[56,57]^ and elevated levels are associated with an increased risk of ALS. The association of these metabolites with an increased risk of ALS suggests that they may be involved in the pathogenesis of ALS by affecting energy metabolism, oxidative stress, and other pathological mechanisms. Their specific mechanisms of action require further study.

Our study provides a scientific basis for future exploration of (N(1) + N(8))-acetylspermidine, Betaine, *N,N*-dimethylalanine, C14:2-DC, Behenylcarnitine (C22), and Deoxycarnitine as potential therapeutic targets for ALS. It emphasizes the need for in-depth research on their mechanisms of action in ALS. These studies will help to provide a more comprehensive understanding of the complex pathogenesis of ALS and provide a scientific basis for developing new treatment strategies. Regarding another part of our study, neuroimaging evidence suggests that changes in cerebral volume may be a secondary phenotype of the pathological process of ALS rather than a causal mediator,^[58]^ which is consistent with currently known studies.^[59-62]^ This finding suggests that the association between metabolomic characteristics and the pathogenesis of ALS may be independent of brain structural changes or that cerebral volume plays only a secondary regulatory role in this causal chain. From a neuropathological perspective, ALS, as a multisystem degenerative disease involving the motor cortex, sensory association area, and prefrontal cortex,^[63]^ may have neuroinflammation, oxidative stress, and autophagy, and other pathways that affect the disease outcome, as follows: branched-chain amino acid metabolites may regulate nuclear factor E2-related factor 2/antioxidant response elements (Nrf2/ARE) signaling pathway to affect intracellular reactive oxygen species (ROS) levels,^[64-67]^ tryptophan metabolic derivatives may act on microglial Toll-like receptor 4 (TLR4) receptors to regulate neuroinflammatory cascades,^[68-71]^ lipid peroxidation products may interfere with molecular chaperone-mediated autophagy and cause abnormal aggregation of TAR DNA binding protein 43 (TDP-43) protein.^[72-74]^ These mechanisms constitute the molecular basis for metabolic imbalances that cause neurodegenerative diseases. Although cerebral volume has not yet been shown to be a mediating factor, multiple undetermined mediating variables, such as neuroinflammation, oxidative stress, and autophagy, reveal a causal path between metabolites and ALS. These findings provide new directions for future research, which can further explore the direct mechanism of the effect of metabolites on ALS. For example, it is possible to verify the effects of metabolites on neuron cells through cell experiments or animal models. Exploring mediating factors by building conditional knockout animal models is also possible, thereby clarifying the neuroprotective mechanisms of specific metabolic pathways.

Admittedly, some limitations should be recognized when interpreting the results of this study. Although we used the largest metabolite GWAS pooled analysis reported to date, the relatively small number of subjects and sample size in the metabolite GWAS may have limited the statistical power of the study, as genetic factors can only explain a small portion of the metabolite’s characteristics, failing to detect significant mediating effects and limited statistical power in detecting causality. Consistent with previous MR studies of microbiome, we used a relatively loose *P*-value threshold to select genetic instrumental variables, which may have introduced weak tool variable bias due to the limited number of mutations associated with metabolites. We tested the strength of instrumental variables and found that the *F* statistic for all instrumental variables exceeded 10, which is the traditional threshold for strong instrumental variables. Third, the exposure factor in this study was plasma metabolites, which reflect systemic metabolic status but may not fully represent the metabolism of the central nervous system. The concentrations of specific metabolites in plasma may significantly differ from those in cerebrospinal fluid. In addition, the blood–brain barrier restricts the entry of specific macromolecules and metabolites from the plasma into brain tissue. Therefore, metabolites in plasma may not fully reflect the metabolic changes in the brain. Metabolites in peripheral plasma may be affected by various factors, including diet, lifestyle, and inflammatory state. Although this study excluded some confounding factors, there may be unknown or missing factors that cause metabolic changes in the blood and central nervous system to not be wholly consistent, all of which may lead to underestimation or misclassification of metabolic changes associated with ALS. Finally, although we analyzed cerebral volume as a possible mediator of the relationship between metabolites and ALS, no significant conclusions were drawn. This study mainly involved individuals of European descent, which suggests that further research is needed to determine whether these results can be generalized to other populations.

## 6. Conclusions

In conclusion, this 2-sample MR study identified 6 metabolites that were genetically associated with ALS risk. Further mediation analysis suggested that cerebral volume was unlikely to be an intermediate factor linking these metabolites with ALS susceptibility. These findings provide additional evidence regarding the potential involvement of circulating metabolites in ALS and warrant further investigation into the underlying biological mechanisms.

## Author contributions

**Conceptualization:** Youjia Liu, Sihui Chen, Yanyun Wu, Wenting Zhou, Huifang Shang.

**Data curation:** Youjia Liu, Wenting Zhou.

**Formal analysis:** Yanyun Wu.

**Visualization:** Ruwei Ou.

**Writing – original draft:** Youjia Liu, Wenting Zhou, Huifang Shang, Ruwei Ou.

**Writing – review & editing:** Youjia Liu, Ruwei Ou.
















